# The role of the TGF-β/LIF signaling pathway mediated by SMADs during the cyst formation of Echinococcus in young children

**DOI:** 10.1186/s12860-022-00452-3

**Published:** 2022-11-28

**Authors:** Shuang-li Qin, Yun Guo, Shui-Xue Li, Ling Zhou, Azguli Maimaiti, Yusufu Akemu, Jun He, Hai-Xia Yao

**Affiliations:** 1 Department of pediatric surgery, people’s Hospital, No. 91, Tianchi Road, Tianshan District, Urumqi, Xinjiang, 830000 Xinjiang Uygur Autonomous Region China; 2 Department of general surgery, Children’s Hospital, Xinjiang, 830000 Uygur Autonomous Region China

**Keywords:** Pediatric hydatidosis, Parasite cyst formation process, TGF-β/Smads/leukemia inhibitory factors (LIF) signaling pathway

## Abstract

**Objective:**

The present study aims to explore the correlation of the transforming growth factor β (TGF-β), drosophila mothers against decapentaplegic protein gene (SMAD) 2/3/4, and leukemia inhibitory factors (LIF) with the cyst formation of hepatic *Echinococcus granulosus* in young children.

**Methods:**

A total of 40 patients who met the diagnostic criteria for children's hydatid disease in people's Hospital of Xinjiang Uygur Autonomous Region between January 2020 and June 2021 were enrolled a s the study subjects. The cystic fluid of these children was collected as the case group and the corresponding infected viscera or pericystic tissue as the control group, with 40 cases in each group. In vitro cultured protoscolice of hydatid cyst, four groups including control group, LIF siRNA group, LIF factor group and SMAD4 siRNA group were divided by inhibiting TGF-β/SMADs signal pathway. Each assay was performed in triplicate. The expression of TGF-β, SMAD2/3/4 and LIF were detected.

**Results:**

The results of the clinical trial showed that the contents of SMAD2 and SMAD3 were increased in the case group compared with the control group; the differences were statistically significant (*P* < 0.05). The expression levels of TGF-β, Smad4, and LIF increased in the case group compared with the control group; however, the differences were not statistically significant. The results of further *in vitro* experiments, the expression levels of TGF-β, SMAD 2/3/4, and LIF after adding siRNA to interfere with Smad4 decreased in the case group compared with the control group; the differences were statistically significant (*P* < 0.05). Compared with the control group, the expression levels of TGF-β, SMAD2/3/4, and LIF increased after treatment with added LIF in the case group, and the expression levels of TGF-β, SMAD2/3/4, and LIF decreased after adding siRNA to interfere with LIF in the case group; the differences were all statistically significant (*P* < 0.05).

**Conclusion:**

SMAD2 and SMAD3 have a certain clinical relevance with hydatidosis in young children. The LIF expression level may be related to the cystic transformation of protoscoleces. It has been suggested that the TGF-β/Smads/LIF signaling pathway may be present in the process of protoscoleces cyst formation; this provides a research basis for the prevention and treatment of post-infection parasitism of E. multilocularis eggs in young children.

## Introduction

Hydatidosis or echinococcosis is a disease caused by the larva of *Echinococcus granulosus* (E. granulosus) infecting the human body [1]. In Northwest China, especially in large pastoral areas (such as Xinjiang and Tibet), children are infected with cystic echinococcosis at a high prevalence, seriously endangering their health. Large hydatid cysts are common in the detection of hydatidosis in children, and the complication rate of cyst rupture is higher than in adults [2, 3]. At present, the regulation mechanism of *Echinococcus multilocularis* (E. multilocularis) eggs forming cysts in a human host after infection is unclear. Relevant studies revealed that there was a complex molecular regulation mechanism in the process from the infection of E. multilocularis eggs to the successful parasitic development, and there was a close mutual response between the host’s body and the larvae. Furthermore, the transforming growth factor β (TGF-β) played a crucial role in these interactive responses [4–6]. However, at present, studies on hydatidosis are mostly focused on adults, and there are few reports on its occurrence in children. It is unclear whether children’s hydatidosis contains the same molecular regulation mechanism. Therefore, the TGF-β/LIF signaling pathway was chosen as the research object in this study in order to conduct a clinical trial and *in vitro* experiments to further explore the role of this signaling pathway in the *in vivo* cyst formation process after the infection of E. multilocularis eggs in children; this is done with the aim to (1) reveal the relevant molecular mechanism of the cyst formation process of hydatidosis parasites in children and (2) hopefully provide a research basis for the prevention and treatment of cystic echinococcosis in children.

## Data and methods

### Clinical study

#### General information

After obtaining the approval of the ethics committee of people's Hospital of Xinjiang Uygur Autonomous Region and the signed informed consent of the children's parents, the cystic fluid (20 ml) and the corresponding paracystal tissues of infected viscera or tissues were collected from children with hydatidosis who met the diagnostic criteria [7] of pediatric hydatidosis in our hospital from January 2020 to June 2021. The paracystal tissues of infected viscera or tissues were used as the control group, and the cystic fluid was used as the case group (*n* = 40 each). All operations were performed with the consent of the children and their families, and relevant informed documents were signed.

#### Experimental methods

##### Sample processing

The tissues were washed with pre-cooled phosphate-buffered saline (PBS), cut into pieces, and weighed. The small pieces of tissue were mixed with the corresponding volume of PBS (generally at the weight/volume ratio of 1:9) and fully ground.

##### Experimental steps

Blank wells (blank control wells without samples and enzyme-labeled reagents) and wells for the sample to be tested were set, respectively. A volume of 40 μl of sample diluent was added into the wells for the sample to be tested on the enzyme-labeled coating plate; next, 10 μl of the sample to be tested was added (the final dilution of the sample was 5×). After sealing the plate with plate sealing membrane, it was incubated at 37°C for 30 minutes. The 30× concentrated washing solution was diluted with distilled water 30× and set aside. The sealing membrane was carefully removed, the liquid was discarded, and the plate was swayed for drying. Each well was filled with detergent, left standing for 30 seconds, and discarded; this procedure was repeated 5×, then the plate was patted dry.

A volume of 50 μL of enzyme-labeled reagent was added to each well, with the exception of the blank well. The plate was incubated at a warm temperature again and washed. Each well was first added with 50 μL of developer A, then with 50 μL of developer B, and was then slightly shaken to mix; the contents then underwent color development at 37°C in the dark for 10 minutes. Each well was added with 50 μL of termination solution, and the reaction was stopped after the blue turned yellow. Zero adjustments were carried out based on the blank well, and the absorbance (optic density) of each well was measured at 450 nm of wavelength according to the number. The measurements were all carried out within 15 minutes after the termination solution was added.

#### Test indexes

The levels of TGF-β, SMAD2, SMAD3, SMAD4, and LIF in the cystic fluid of children with hydatidosis and the corresponding paracystal tissues of infected viscera or tissues were measured by the enzyme-linked immunosorbent assay (ELISA).

### *In vitro* study

#### *In vitro* culture and grouping of protoscoleces

The method of Liying Yuan [8] was adopted to digest, separate, and culture protoscoleces obtained from the inner cyst of the hydatid cyst in children with parasitic Echinococcus. The activity of the protoscoleces was detected using 0.1% eosin staining. Protoscoleces with >90% activity were used for testing. Four groups were established for this experiment: the control group (control), the LIF siRNA group (LIF siRNA), the LIF factor group (LIF factor), and the Smad4 siRNA group (SMAD4 siRNA). Each group was triplicate.

#### Experimental methods

The isolated protoscoleces cultured in a six-well plate were observed. Under the visual field, the number, proportion, and activity of protoscoleces with cystic characteristics were examined and recorded. The protoscoleces were treated with LIF, LIF siRNA interference, and SMAD4 siRNA interference, respectively. After the treatment, the number, proportion, and activity of protoscoleces with cystic characteristics were examined under the visual field and recorded. After microscopic examination, the cultured protoscoleces of each group were killed, and the total protein was extracted. The expression of LIF was detected by ELISA. The correlation between LIF expression after interference and the regulation of protoscoleces cyst formation was statistically analyzed.

#### Test indexes

Expression levels of TGF-β, SMAD2, SMAD3, SMAD4, and LIF in the samples of Echinococcus Taenia protoscoleces of each group were detected using ELISA. The ELISA method was the same as in the clinical experiment.

### Statistical methods

Data were statistically analyzed using the SPSS 17.0 software, and statistical analysis and charting were conducted using the GraphPad Prism 7.00 software. In the comparison of the clinical trial data, measurement data were expressed as mean ± standard deviation ($\overline{\mathrm x}$ ± SD), and count data were expressed as percentages (%); comparisons were made using the t-test and rank-sum test, respectively. The data obtained from the *in vitro* experiment were compared among groups using a one-way analysis of variance (*F* test). The inspection level was set at α = 0.05.

## Results

### Clinical trials

#### Expression levels of TGF-β, SMAD2, SMAD3, SMAD4, and LIF

**Fig. 1:**
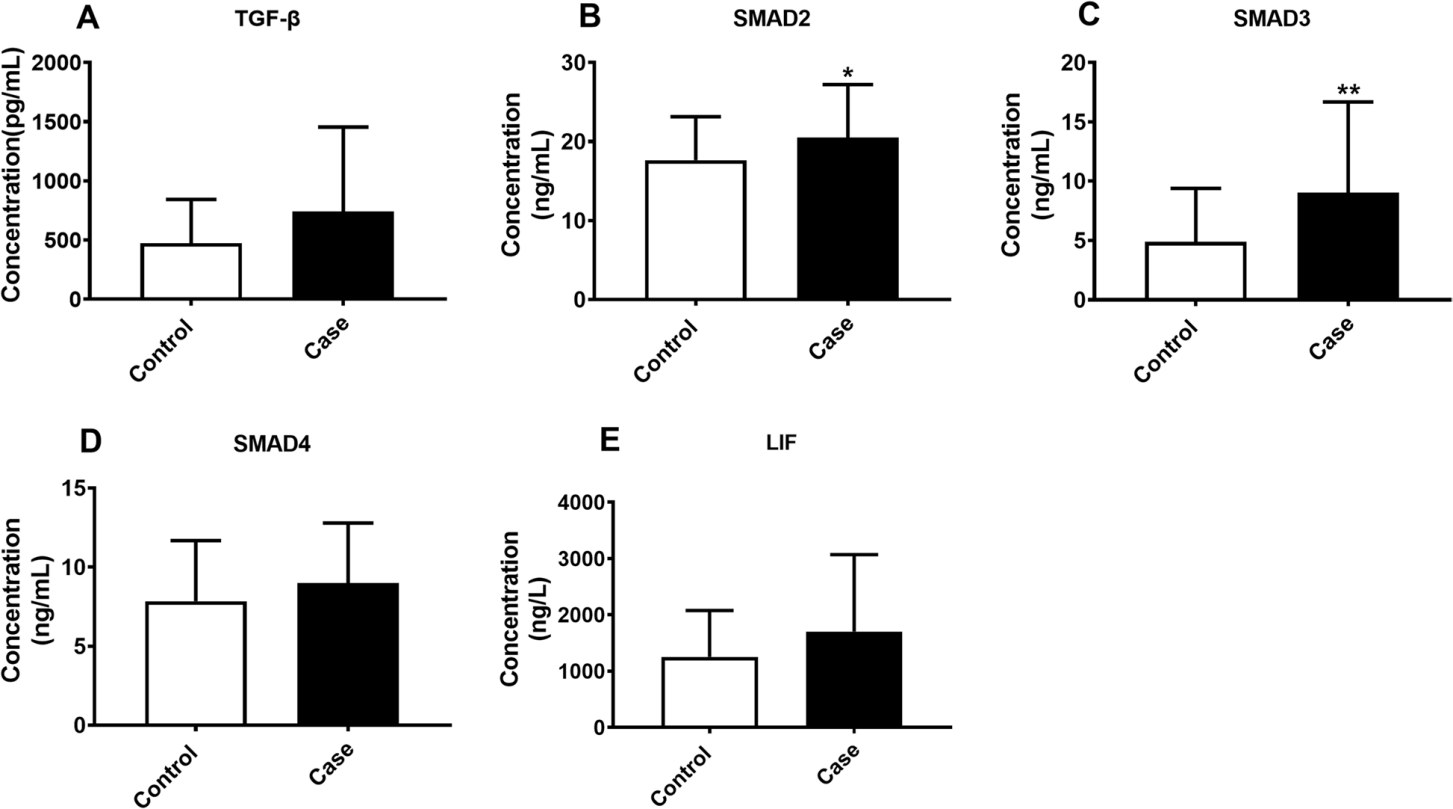
TGF-β, Expression of Smad2, Smad3, Smad4 and LIF

The expression levels of TGF-β, SMAD2, SMAD3, SMAD4, and leukemia inhibitory factors (LIF) in the cystic fluid and the corresponding paracystal tissues of infected viscera or tissues in children with hydatidosis were measured using the ELISA kit. The contents of SMAD2 and SMAD3 increased in the case group compared with the control group; the differences were statistically significant (*P* < 0.05, *P* < 0.01). The TGF-β, SMAD4, and LIF expression levels increased in the case group; however, the differences were not statistically significant (Table 1 and Fig. 1).

**Table 1:** Expression levels of TGF-β, SMAD2, SMAD3, SMAD4 and LIF ($\overline{\mathrm x}$ ± SD)

Group	TGF-β (pg/mL)	SMAD2 (ng/mL)	SMAD3 (ng/mL)	SMAD4 (ng/mL)	LIF (ng/L)
Control group (*n*=40)	473.3 ± 369.5	17.63 ± 5.51	4.885 ± 4.51	7.837 ± 3.83	1253 ± 824.1
Case group (*n*=40)	740.6 ± 713.9	20.49 ± 6.728	9.041 ± 7.64	8.975 ± 3.813	1703± 1368
*Z/t*	-1.366	2.076	-2.771	-0.808	-1.299
*P*	0.172	0.0412	0.006	0.419	0.194

### *In vitro* experiment

#### Expression levels of TGF-β, SMAD2, SMAD3, SMAD4, and LIF

**Fig. 2:**
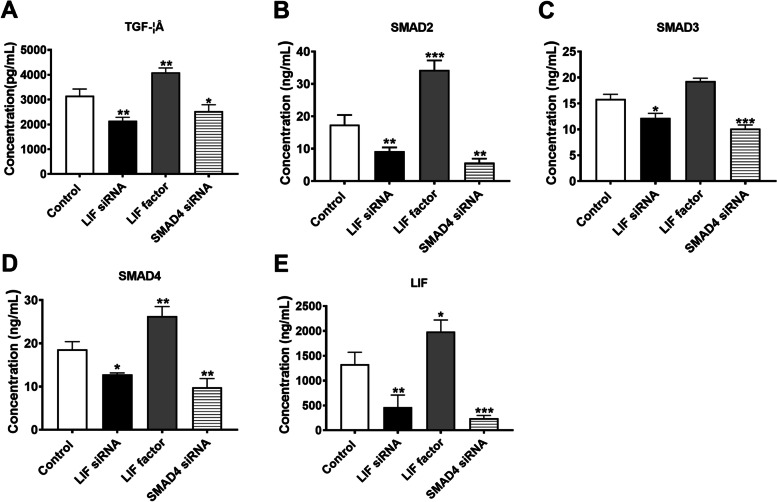
TGF-β, Expression of Smad2, Smad3, Smad4 and LIF **A**TGF-β Expression quantity; **B** Smad2 expression; **C** Smad3 expression; **D** Smad4 expression; **E** LIF expression

After adding siRNA to interfere with SMAD4, the expression levels of TGF-β, SMAD2/3/4, and LIF decreased in the case group compared with the control group; the differences were statistically significant (*P* < 0.05, *P* < 0.01, *P* < 0.001). The expression levels of TGF-β, SMAD2/3/4, and LIF increased after treatment with added LIF in the case group compared with the control group; the differences were statistically significant (*P* < 0.01, *P* < 0.001, *P* < 0.05). After adding siRNA to interfere with LIF, the expression levels of TGF-β, SMAD2/3/4, and LIF decreased in the case group compared with the control group; the differences were statistically significant (*P* < 0.01, *P* < 0.05) (Table 2 and Fig. 2).

**Table 2:** Expression levels of TGF-β, SMAD2, SMAD3, SMAD4 and LIF ($\overline{\mathrm x}$± SD)

Group	TGF-β (pg/mL)	SMAD2(ng/mL)	SMAD3(ng/mL)	SMAD4(ng/mL)	LIF (ng/mL)
Control group (*n*=3)	3152 ± 275.5	17.43 ± 2.992	15.86 ± 0.8953	18.61 ± 1.797	1334 ± 235.8
LIF siRNA group (*n*=3)	2151 ± 136.3	9.237 ± 1.173	12.22 ± 0.8493	12.80 ± 0.3624	464.3 ± 244.3
LIF group (*n*=3)	4100 ± 173.0	34.3 ± 2.975	19.29 ± 0.568	26.32 ± 2.201	1984 ± 236.5
SMAD4 siRNA group (*n*=3)	2529 ± 266.8	5.682 ± 1.271	10.12 ±0.7136	9.80 ± 2.042	245.6 ± 52.66
*F*	44.5	93.76	83.39	51.33	44.52
*P*	0.0001	0.0001	0.0001	0.0001	0.0001

## Discussion

### Characteristics and hazards of hydatidosis in young children

(1) As the liver tissue of young children has a loose structure and is active in development, it provides a relatively good living environment for the activation of E. granulosus eggs [7]; eggs can be activated and can grow rapidly after entering the hepatic portal vein through the intestinal mucosa. (2) A hydatid cyst in young children is in the early cyst stage, with a high activity of cyst fluid and great tension of the cyst wall, which is prone to rupture and infection. (3) The morphological characteristics of hepatic cystic *Echinococcosis* in young children are that the cyst grows faster than in adults, the cyst wall is thin, and the infection often simultaneously occurs in both the liver and the lung. (4) Due to the poor self-protection awareness of young children, the probability of intraperitoneal implantation due to the rupture of the hydatid cyst is higher than in adults, and the cure is difficult [9]. In summary, after the formation of a hepatic hydatid cyst in children, they are either surgically removed or eventually rupture, resulting in abdominal implantation; this causes serious trauma to the child’s physical and mental health. Blocking the cyst formation in the early stages in the early stage after the hydatid eggs enter the children’s body is very important for the treatment of children’s hydatidosis.

### TGF-β/ Smads/LIF signaling pathway

A large number of previous studies revealed that serum expression levels of TGF-α and TGF-β were significantly higher in patients with hydatidosis than in non-patients, and the expression levels of TGF-α and TGF-β in cystic tissue effusion were significantly higher than in paracystal tissue or the outer capsule in patients. Researchers also found through studies on the inflammatory factors of alveolar hepatic hydatidosis that TGF-β and other cytokines played important roles in the progression of hepatic hydatidosis [4–6]. Leukemia inhibitory factors (LIF) are a cytokine with multiple functions [10]. The most important discovery is that LIF can maintain the pluripotency of mouse embryonic stem cells [11, 12]. The loss of cell pluripotency is a necessary process during the development and maturation of the body, and cell pluripotency is gradually lost with the degree of cell differentiation and the maturity of body development. Only a few cells in a mature organism can maintain pluripotency. In fact, TGF-β is not only an important anti-inflammatory factor but also a very important transforming growth factor in the body; it regulates important signaling pathways in cells. The regulatory network that TGF-β participates in within the body is very complex. The members of Smads protein family in the pathway mainly include SMAD2, SMAD3 and SMAD4. The SMAD protein is a very important transcription regulatory factor of the TGF-β superfamily; it can regulate the expression of many factors in cells, one of which is LIF. Previous studies revealed that when Smad4 or SMAD2/3 is interfered in by siRNA, the intracellular expression of LIF induced by TGF-β will be significantly inhibited [13–15].

### Pathological and physiological differences between cystic fluid and paracystic tissue

Echinococcus contains many protoscolice (scolex). Chen et al. [16] stimulated spleen cells of mice with protoscolice and hydatid cyst fluid respectively, and the results showed that the increase of TGF-β is consistent at the protein level and the molecular level, thus affecting the immune escape from the host. Zhou et al. [17] stimulated mouse spleen cells with hydatid cyst fluid, The increase of SMAD4, the downstream signal transduction protein of TGF-β, suggests that the differentiation of Treg cells and the SMAD4 signal pathway may be involved in the immune escape process of the cyst fluid to the host, which is consistent with the results of this study in vitro.

Therefore, we speculate that the SMAD pathway in pericystic tissues (hepatocytes) is inactive under normal conditions, and can specifically activate TGF/Smads signal pathway under the stimulation of hydatid cyst stimulates Stimulate the phosphorylation of TGF-β receptors distributed on the liver cell membrane, and then induce the expression of receptor regulated SMAD2/3/4 in the cytoplasm to increase, activate LIF factor. The SMAD protein transmits the pathogenic signal into the nucleus, and the corresponding target genes in the nucleus are activated and transcribed, leading to pathological liver injury. The host's immune ability may be down regulated, thus avoiding the host's immune attack.

### Analysis of study results

In the clinical part of this study, the levels of SMAD2 and SMAD3 in the cystic fluid were significantly higher in the case group than in the control group; the differences were statistically significant (*P* < 0.05, *P* < 0.01). Further *in vitro* experiment verified that after adding siRNA to interfere with SMAD4, the expression levels of TGF-β, SMAD 2/3/4, and LIF decreased in the case group compared with the control group; the differences were statistically significant (*P* < 0.05). Compared with the control group, the expression levels of TGF-β, SMAD2/3/4, and LIF increased after treatment with added LIF in the case group. After adding siRNA to interfere with LIF, the expression levels of TGF-β, SMAD2/3/4, and LIF decreased in the case group compared with the control group; the differences were all statistically significant (*P* < 0.05). Although the expression of TGF-β was not statistically significant in clinical trials, its content in the case group showed an upward trend. The reasons for this are as follows: (1) the sample size was small; (2) in this study, the paracystal tissues of the corresponding infected viscera or tissues in the body of children were greatly interfered in by the factors of different infection states in the host. In future clinical trials, the *in vivo* infection factors in the host need to be further eliminated and the sample size needs to be increased to optimize the experiment. Therefore, it is speculated that in the process of human infection with hydatid eggs in this study, the eggs hatched into larvae and then stopped developing into cysts; during this process, the pluripotency of hydatid cells was partially lost. The key factor that could maintain this pluripotency may be the LIF factor activated by a surging expression of TGF-β. Due to the small sample size of this study, whether it is generalizability and applicability for the general pediatrics population with cystic echinoisis is unclear and still needs to be verified by a large amount of research data.

## Conclusions

The present study suggests that SMAD2 and SMAD3 have certain clinical relevance to hydatidosis in young children. The LIF may be related to the cystic transformation of protoscoleces. It is suggested that the TGF-β/Smads/LIF signaling pathway may be present in the process of protoscoleces cyst formation. This provides a research basis for the prevention and treatment of post-infection parasitism of E. multilocularis eggs in young children.

## Data Availability

The datasets used and/or analysed during the current study available from the corresponding author on reasonable request. We declared that materials described in the manuscript, including all relevant raw data, will be freely available to any scientist wishing to use them for non-commercial purposes, without breaching participant confidentiality.
